# Who Is This Man?

**DOI:** 10.3201/eid1510.090128

**Published:** 2009-10

**Keywords:** Yellow fever, extrinsic incubation period, viruses, discovery, photo quiz

**Figure:**
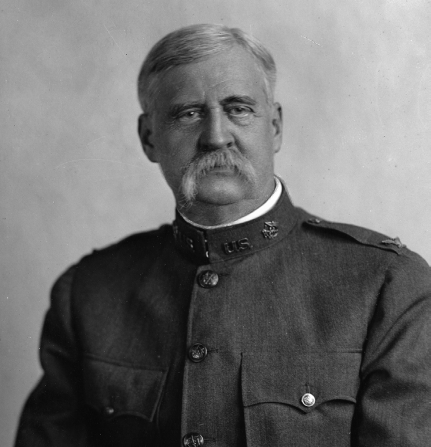
He discovered the extrinsic incubation period of yellow fever.

Here is a clue: He discovered the extrinsic incubation period of yellow fever.

Who is he?
A) Henry Rose Carter
B) Carlos J. Finlay
C) William Crawford Gorgas
D) Jesse William Lazear
E) Walter Reed

Decide first; then turn the page.

